# Mitochondrial haplotypes and microsatellite genotypes for grass and water snakes of the genus *Natrix*: A range-wide resource (Squamata, Natricidae)

**DOI:** 10.3897/BDJ.14.e208590

**Published:** 2026-08-31

**Authors:** Andrea Criado-Flórez, Marika Asztalos, Carolin Kindler, Adrian Neumann, Nadine Schultze, Uwe Fritz

**Affiliations:** 1 Museum of Zoology, Senckenberg Dresden, Dresden, Germany Museum of Zoology, Senckenberg Dresden Dresden Germany; 2 Technical University of Munich, TUM School of Life Sciences, Freising, Germany Technical University of Munich, TUM School of Life Sciences Freising Germany; 3 Institute of Biology, Leipzig University, Leipzig, Germany Institute of Biology, Leipzig University Leipzig Germany

**Keywords:** Database, Palaearctic, phylogeography, Reptilia, Serpentes

## Abstract

**Background:**

Grass and water snakes of the genus *Natrix* are a dominant component of the Palaearctic reptile fauna, with a broad distribution ranging from North Africa across much of Europe to Lake Baikal in Central Asia. As part of a series of studies on the phylogeography and systematics of the group, a large database of mtDNA sequences and microsatellite genotypes was compiled for all five *Natrix* species.

**New information:**

We present a range-wide genetic database comprising georeferenced information for approximately 3,700 snakes belonging to the grass snake complex (i.e. *Natrix astreptophora*, *N. helvetica*, *N. natrix*), *N. maura*, *N. tessellata* and interspecific hybrids. This unique repository can serve as a comprehensive resource for phylogeographic, population genetic and systematic studies, as well as for monitoring hybrid zone dynamics and future anthropogenic hybridisation and admixture resulting from translocated non-native snakes.

## Introduction

Grass and water snakes of the genus *Natrix* are the most abundant snakes across much of their Palaearctic range (Speybroeck et al. 2016). The five *Natrix* species are distributed from North Africa across the Iberian Peninsula and much of Europe to Lake Baikal in Central Asia (Sindaco et al. 2013). During the past decade, a series of papers on the phylogeography and systematics of grass snakes (*Natrix astreptophora*, *N. helvetica*, *N. natrix*) made use of a constantly growing large database of microsatellite genotypes and mtDNA sequences (Kindler et al. 2013, Kindler et al. 2014, Pokrant et al. 2016, Kindler et al. 2017, Kindler and Fritz 2018, Kindler et al. 2018a, Kindler et al. 2018b, Glaw et al. 2019, Schultze et al. 2019, Asztalos et al. 2020, Schultze et al. 2020, Ahnelt et al. 2021, Asztalos et al. 2021a, Asztalos et al. 2021b, Asztalos et al. 2021c, Jablonski et al. 2023a, Jablonski et al. 2023b, Neumann et al. 2024, Criado-Flórez et al. 2025, Criado-Flórez et al. 2026, Griesbaum et al. 2026). In the present paper, we publish the current version of the underlying database, supplemented with mtDNA sequences of grass snakes, viperine snakes (*N. maura*) and dice snakes (*N. tessellata*) from all other available sources for *Natrix* species, mainly from Guicking (2004), Guicking et al. (2006), Guicking et al. (2008), Guicking et al. (2009), Schild et al. (2024) and Simonov et al. (2024); for further references, see Suppl. material 1: Table S1. This georeferenced database comprises mtDNA sequences (cyt *b*, ND4, the latter often plus adjacent DNA coding for tRNAs) and microsatellite genotypes for 3,061 grass snakes, 69 *N. natrix* x *N. tessellata* hybrids and mtDNA sequences for one *N. astreptophora* x *N. maura* hybrid. In addition, it contains mtDNA sequences for 130 *N. maura* and 433 *N. tessellata* and 59 microsatellite genotypes for *N. tessellata*. This dataset provides for all taxa range-wide coverage and constitutes a valuable resource for future population genetic, phylogeographic and taxonomic studies of these snake species.

## General description

### Purpose

The database covers all five *Natrix* species. The dataset (Suppl. material 1: Table S1) reports, for each sample and whenever known, its mitochondrial identity (mtDNA clade and individual cyt *b* and ND4 haplotypes), microsatellite genotype and georeferenced collection site (WGS 84).

### Additional information

For *Natrix maura* and most *N. tessellata*, the dataset contains mitochondrial data only. For the grass snake complex (*N. astreptophora*, *N. helvetica*, *N. natrix*), some *N. tessellata* and *N. natrix* x *N. tessellata* hybrids, genotypes at 13 microsatellite loci are also provided where available. For each individual, the microsatellite genotype is presented as rounded and standardised allele lengths at each locus, using the values of the original sources. Under "References", the original source(s) in which the primary data were first published are listed; the laboratory and data-processing procedures are also described there. With the exception of approximately 200 Swiss samples from Kindler et al. (2017), all microsatellite genotypes were generated in the same laboratory (Senckenberg Dresden). Fragment lengths from the Swiss samples were calibrated as described in Kindler et al. (2017). For grass snakes, the nomenclature of mitochondrial haplotypes follows Kindler et al. (2017) and subsequent studies. For *N. tessellata*, the haplotype nomenclature of Guicking et al. (2009) and Asztalos et al. (2021c) is used; for cyt *b* haplotypes of *N. maura*, that of Guicking et al. (2008). An equivalent nomenclature is applied to ND4 haplotypes and the acronym ALG is introduced for the additional *N. maura* clade from eastern Morocco and Algeria described by Barata et al. (2008). Sampling sites were individually verified using Google Maps and incorrect spellings and coordinates were corrected wherever possible. Our dataset also includes several previously unpublished haplotypes for grass snakes (cyt *b*: c14, c15, c16, gn42, gn43, gn44, gn45, gn46, h51, r87, r88, r89; ND4+tRNAs: c6, c7, r47). The respective sequences were generated as described in Kindler et al. (2013); haplotypes of new sequences were identified using TCS parsimony networks as implemented in Hapsolutely (Vences et al. 2024). GenBank or ENA accession numbers and sequences for all haplotypes are provided in the Suppl. material 2 (Table S2).

**Figure 1.**
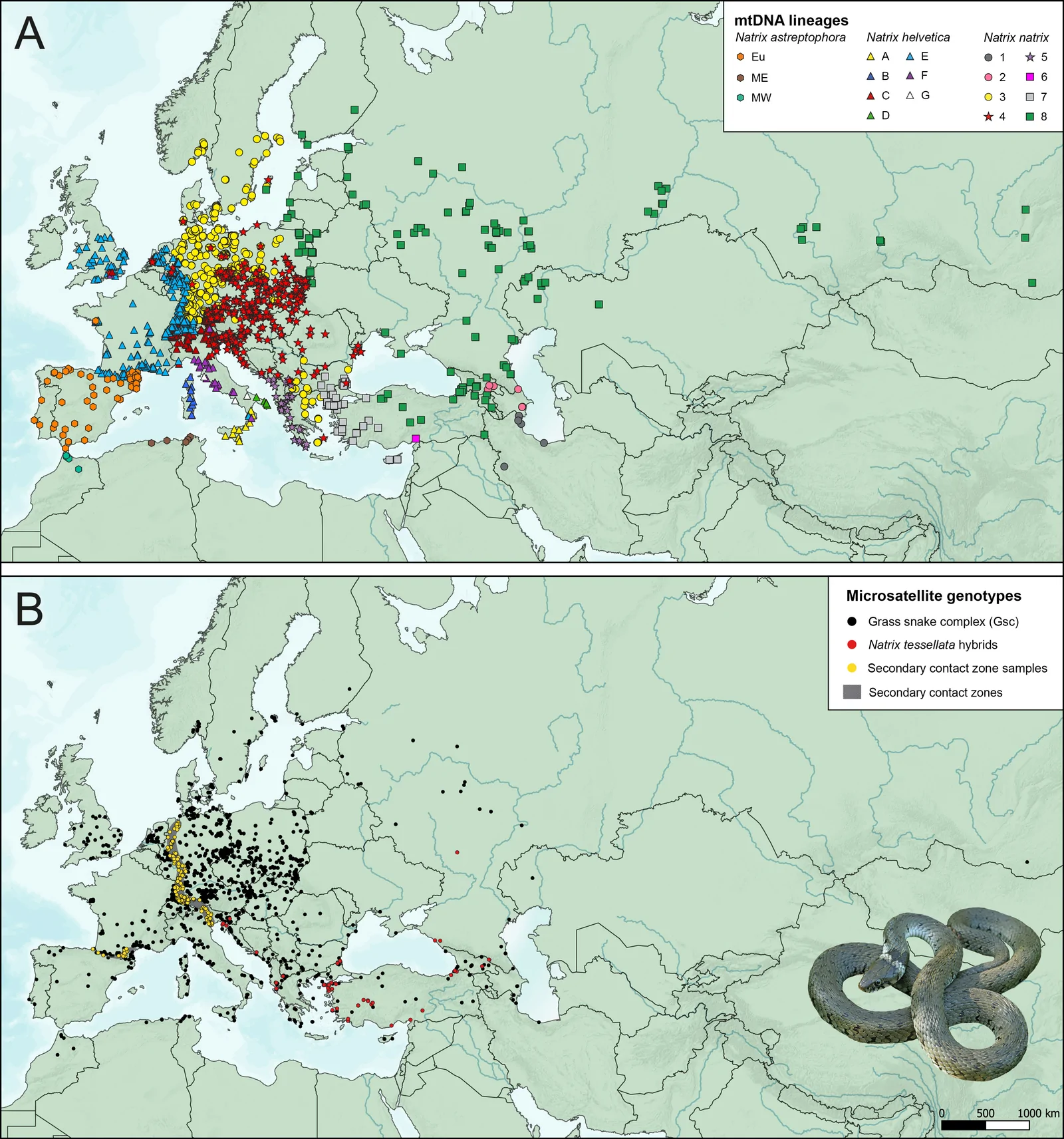
**A** Records of mtDNA lineages within the grass snake complex (*Natrix astreptophora*, *N. helvetica*, *N. natrix*); **B** Collection sites of grass snakes and their hybrids with *Natrix tessellata* for which microsatellite data are available. Inset picture: *N. helvetica*, Campan, Haut-Pyrénées, France (photo: C. Delmas).

**Figure 2.**
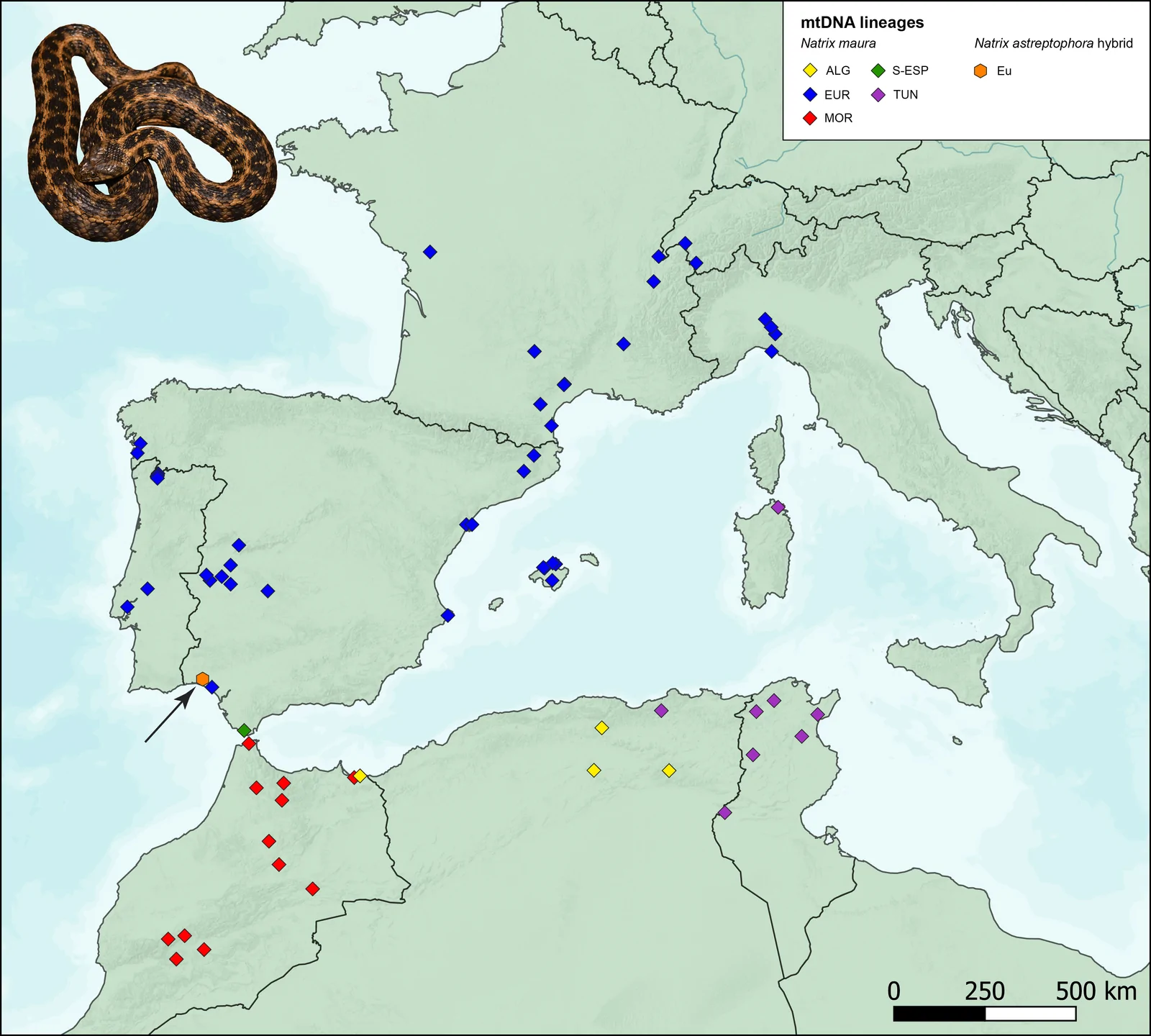
Records of mtDNA lineages of viperine snakes (*Natrix maura*). The *N. astreptophora* haplotype (arrow) corresponds to the *N. astreptophora* x *N. maura* hybrid described in Criado-Flórez et al. (2025). Inset picture: *N. maura*, Rif, Morocco (photo: G. Martínez del Mármol).

**Figure 3.**
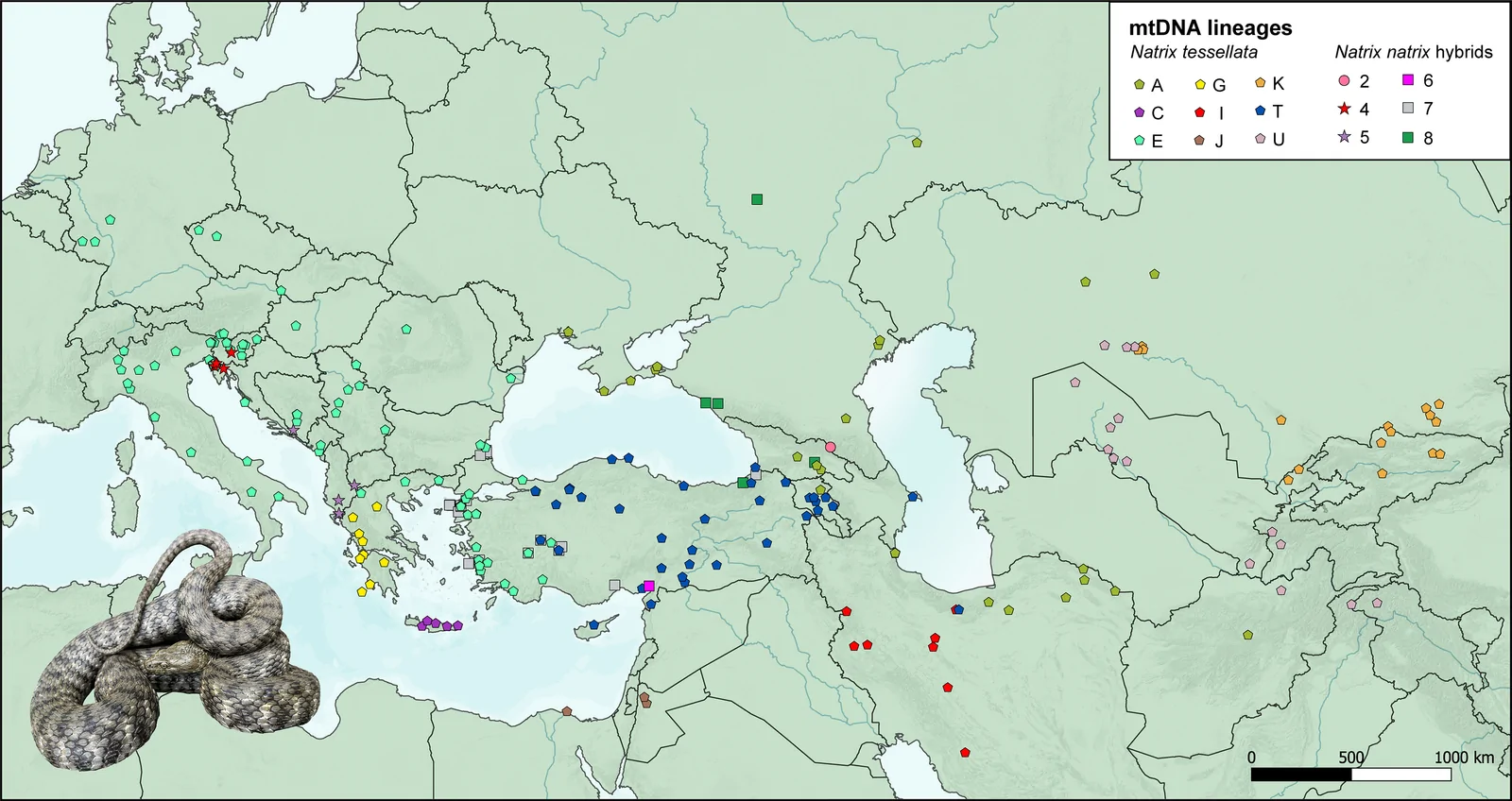
Records of mtDNA lineages of dice snakes (*Natrix tessellata*) and hybrids with *N. natrix*. Shown are hybrids carrying *N. natrix* haplotypes; hybrids carrying *N. tessellata* haplotypes also occur (see Suppl. material 1: Table S1 and Asztalos et al. 2021c). Inset picture: *N. tessellata*, Meran, South Tyrol, Italy (photo: U. Fritz).

The accompanying maps (Figs 1, 2, 3) show the distribution of the mitochondrial lineages of grass snakes and the other two species; for individual haplotypes, see Suppl. material 1 (Table S1). For the grass snake complex, Fig. 1 also depicts the sites of the genotyped samples.

Hybridisation between *N. astreptophora* and *N. maura* and between *N. natrix* and *N. tessellata* is rare (Asztalos et al. 2021c, González de la Vega et al. 2021, Fritz and Ihlow 2022, Criado-Flórez et al. 2025). A single F_1_ hybrid between *N. astreptophora* and *N. maura* has been genetically verified (Criado-Flórez et al. 2025), whereas several F_1_ hybrids as well as backcrosses or later-generation hybrids are known for *N. natrix* x *N. tessellata* (Asztalos et al. 2021c). This allows hybrids to be flagged in our dataset (Suppl. material 1: Table S1), although hybridisation amongst the mentioned species is not restricted to clearly circumscribed geographic regions (Figs 2, 3). In contrast, hybridisation and limited gene flow between *N. astreptophora* and *N. helvetica* and between *N. helvetica* and *N. natrix* are more frequent along narrow geographic contact zones (Kindler et al. 2017, Schultze et al. 2019, Asztalos et al. 2020, Schultze et al. 2020, Asztalos et al. 2021a, Neumann et al. 2024; see our Fig. 1). In the latter two cases, identification of pure individuals and hybrids can vary amongst analyses, depending on the samples included. Therefore and because of prolonged computation time, we did not attempt to classify the samples of these three species using STRUCTURE analyses (Pritchard et al. 2000) and the entire database. Instead, we treat all samples as representing the "grass snake complex" and show in the accompanying maps the extent of the hybrid zones inferred from previous studies. Any sample within these regions may represent either one of the parental species or a hybrid. Beyond the hybrid zones, pure individuals are expected, although translocations may create exceptions. Long-distance translocation of grass snakes and their hybridisation with native individuals are well known, see e.g. Kindler et al. (2017), Asztalos et al. (2020), Ahnelt et al. (2021) and Asztalos et al. (2021b). This is evident in grass snakes from Newdigate (Surrey) and the West Midlands, both Great Britain, and several sites in France, Germany, Italy, the Netherlands and Switzerland, where non-native mitochondrial lineages have been detected (see also the mismatched records in Fig. 1A). At some of these sites, non-native or admixed populations have become established, threatening the genetic integrity of local grass snakes (Kindler et al. 2017, Schultze et al. 2020, van Riemsdijk et al. 2020, Ahnelt et al. 2021, Asztalos et al. 2021b, Griesbaum and Pacher 2024, Schild et al. 2024, Griesbaum et al. 2026). Our database allows both tracking population dynamics in natural hybrid zones and detecting future anthropogenic admixture beyond these zones.

## Geographic coverage

### Description

Palaearctic.

## Usage licence

### Usage licence

Open Data Commons Attribution License

## Data resources

### Data package title

Supporting Tables S1, S2. 

### Number of data sets

2

### Data set 1.

#### Data set name

Table S1.

#### Data format

Excel

#### Data format version

xlsx

#### Description

Database containing mtDNA haplotypes and microsatellite genotypes of *Natrix* species (release date: 1 August 2026). Short mtDNA sequences allowing only mitochondrial lineage assignment, not individual haplotype determination, marked with asterisks. Missing data are indicated by dashes.

### Data set 2.

#### Data set name

Table S2.

#### Data format

Excel

#### Data format version

xlsx

#### Description

Individual haplotypes for the mitochondrial cyt *b* gene and the ND4 gene plus adjacent DNA coding for tRNAs in *Natrix* species (release date: 1 August 2026).

## Supplementary Material

EDA2D82D-7B41-5E92-9281-9693DDDC5100 10.3897/BDJ.14.e208590.suppl1 Supplementary material 1 Table S1 Data type xlsx Brief description Database containing mtDNA haplotypes and microsatellite genotypes of *Natrix* species (release date: 1 August 2026). Short mtDNA sequences allowing only mitochondrial lineage assignment, not individual haplotype determination, marked with asterisks. Missing data are indicated by dashes. File: oo_1758240.xlsx https://binary.pensoft.net/file/1758240 Andrea Criado-Flórez, Marika Asztalos, Carolin Kindler, Adrian Neumann, Nadine Schultze, Uwe Fritz

7D1C0F59-193C-5844-A262-9D56FECF4D6E 10.3897/BDJ.14.e208590.suppl2 Supplementary material 2 Table S2 Data type xlsx Brief description Individual haplotypes for the mitochondrial cyt *b* gene and the ND4 gene plus adjacent DNA coding for tRNAs in *Natrix* species (release date: 1 August 2026). File: oo_1744791.xlsx https://binary.pensoft.net/file/1744791 Andrea Criado-Flórez, Marika Asztalos, Carolin Kindler, Adrian Neumann, Nadine Schultze, Uwe Fritz
